# Genetic Modifiers of Duchenne Muscular Dystrophy in Chinese Patients

**DOI:** 10.3389/fneur.2020.00721

**Published:** 2020-07-29

**Authors:** Menglong Chen, Liang Wang, Yaqin Li, Yongjun Chen, Huili Zhang, Yuling Zhu, Ruojie He, Huan Li, Jinfu Lin, Yu Zhang, Cheng Zhang

**Affiliations:** ^1^Department of Neurology, The First Affiliated Hospital, Sun Yat-sen University, Guangzhou, China; ^2^Guangdong Provincial Key Laboratory of Diagnosis and Treatment of Major Neurological Diseases, National Key Clinical Department and Key Discipline of Neurology, Guangzhou, China; ^3^Department of Neurology, The First Affiliated Hospital, Jinan University, Guangzhou, China; ^4^Department of Neurology, The Seventh Affiliated Hospital, Sun Yat-sen University, Shenzhen, China; ^5^Department of Neurology, Nanhua Hospital Affiliated to Nanhua University, Hengyang, China; ^6^Department of Neurology, Guangzhou First People's Hospital, South China University of Technology, Guangzhou, China

**Keywords:** Duchenne muscular dystrophy, genetic modifiers, single nucleotide polymorphisms, *SPP1*, *LTBP4*

## Abstract

**Background:** Duchenne muscular dystrophy (DMD) is a fatal, X-linked recessive muscle disorder characterized by heterogeneous progression and severity. We aimed to study the effects of single nucleotide polymorphisms (SNPs) in *SPP1* and *LTBP4* on DMD progression in Chinese patients.

**Methods:** We genotyped *LTBP4* haplotypes and the *SPP1* promoter SNPs rs28357094, rs11730582, and rs17524488 in 326 patients registered in the neuromuscular database of The First Affiliated Hospital of Sun Yat-sen University. Kaplan-Meier curves and log-rank tests were used to estimate and compare median age at loss of ambulation, while Cox proportional hazard regression models were used as to analyze the effects of glucocorticoids treatments, *DMD* genotype, and *SPP1*/*LTBP4* SNPs on loss of ambulation.

**Results:** The CC/CT genotype at rs11730582 was associated with a 1.33-year delay in ambulation loss (*p* = 0.006), with hazard ratio 0.63 (*p* = 0.008), in patients with truncated *DMD* genotype and undergoing steroid treatment. On the other hand, rs17524488 in *SPP1* and the IAAM/IAAM haplotype in *LTBP4* were not associated with time to ambulation loss.

**Conclusions:** *SPP1* rs11730582 is a genetic modifier of the long-term effects of steroid treatment in Chinese DMD patients. Thus, any future clinical study in DMD should adjust for glucocorticoids use, *DMD* genotype, and *SPP1* polymorphisms.

## Introduction

Duchenne muscular dystrophy (DMD) is a fatal, X-linked recessive muscle disease caused by a spectrum of mutations in the *DMD* gene that result in the loss of dystrophin from sarcolemma (1). Although the underlying molecular defect, i.e., complete deficiency of dystrophin, is homogeneous, disease progression is not (2). The variability in phenotype severity and clinical course indicates that other factors may be involved, including genetic modifiers beyond *DMD* gene or environmental factors such as glucocorticoids (GCs) treatment and physical therapy (3–6). The single nucleotide polymorphism (SNP) rs28357094 (-66T/G) in the *SPP1* promoter was identified to be one such genetic modifier in several studies (7–9). In particular, a rare G allele at this site reduces *SPP1* transcription, and is associated with earlier loss of ambulation. Two other common SNPs, rs11730582 (-443C/T) and rs17524488 (-156G/GG), also clearly impact the activity of the *SPP1* promoter, and are often investigated with rs28357094 as genetic modifiers of several diseases (10–15). Whether these SNPs affect the progression of DMD remains unknown, although SPP1, also known as osteopontin, is a cytokine that regulates inflammation, tissue remodeling, cellular immunity, and tumor cell metastasis in many pathologies and disorders (16, 17). Notably, SPP1 expression is elevated in muscles in patients with DMD (18, 19), as well as in dystrophin-deficient mice (20). Remarkably, SPP1/dystrophin double-mutant mice present reduced inflammation, less fibrosis, and increased muscle strength, suggesting that ablation of SPP1 protects the dystrophic muscle (21). Besides, the IAAM haplotype of *LTBP4*, consisting of the four non-synonymous SNPs rs2303729, rs1131620, rs1051303, and rs10880, is associated with prolonged ambulation (9, 22, 23). This haplotype is believed to accelerate muscle regeneration and alleviate muscle fibrosis by reducing TGF-β signaling (22).

DMD and its milder allelic form, Becker muscular dystrophy (BMD), mostly conform to the reading frame rule, in which the former is caused by frameshift mutations, while the latter is due to in-frame mutations (24). However, 4–15% of DMD and 7–37% of BMD cases did not conform to this rule in our previous study and other (25, 26), and were believed to be due to the rescue of a small amount of dystrophin despite frameshift mutations, or to the deletion of essential domains in dystrophin despite in-frame mutations. For instance, patients with nonsense mutations in in-frame exons (27) or deletions amenable to endogenous skipping of exon 44 (28, 29) seem to experience prolonged ambulation, while patients with in-frame mutations starting in exon 3 present more serious phenotypes (25).

*SPP1* rs28357094 and *LTBP4* haplotypes have been surveyed in several cohorts, but not in Chinese patients, and results remain controversial. Considering the massive caseload in China, significant genetic modifiers should be identified to properly interpret clinical studies of the disease in the country. Thus, we assessed the impact of *LTBP4* haplotypes and the *SPP1* SNPs s28357094, rs11730582, and rs17524488 on ambulation loss in patients registered with the neuromuscular database of First Affiliated Hospital of Sun Yat-sen University, controlling for *DMD* genotype and steroid treatment. The database consists of 1,290 male patients with Duchenne or Becker muscular dystrophy, all patients in the database had typical symptoms and signs of muscular dystrophy such as unable to run and jump, waddling gait, limb weakness, or amyotrophy, Gower sign (+) and gastrocnemius muscle pseudohypertrophy, most of the suspected cases were identified by genetic test for *DMD* gene and muscle pathology. For financial reasons, a small proportion of patients received only muscle pathology tests which is the gold standard in diagnosing DMD (25).

## Materials and Methods

### Patients and Inclusion Criteria

Patients aged 5–20 years old were initially included. As loss of ambulation is widely considered to be an important milestone of disease course, and is usually recalled with precision by patients and/or their families, non-ambulant patients were prior selected. We defined loss of ambulation as continuous wheelchair dependence, and estimated the age at loss of ambulation to the nearest month. And we also confirmed loss of ambulation as the inability to continuously walk unassisted for 10 m. Subsequently, patients who were unable to provide DNA samples or accurate information on GCs treatments and ambulation status were excluded. In the end, the cohort consisted of 326 patients. This study was approved by the ICE for Clinical Research and Animal Trial at First Affiliated Hospital of Sun Yat-sen University, and informed consent was obtained from each included patient prior to the survey.

### Stratification

To eliminate confounding effects, patients were stratified by GCs treatment. Patients who had received continuous treatment for at least 1 year were deemed to have been treated. This standard was selected in light of the clinical consensus that long-term steroid use indeed delays disease progression. All other patients were considered untreated. Patients were further stratified by primary *DMD* mutation. Non-truncated mutations included deletions amenable to skipping exon 44, deletion of exons 3–7, missense mutations, in-frame deletions or duplications, and nonsense mutations, deletions, or insertions within in-frame exons according to previous study (27, 29–32). All other mutations were considered truncated mutations. We note that the relatively broad definition of a non-truncation mutation led to a more conservative analysis of the effect of *SPP1* and *LTBP4* genotypes.

### *SPP1* and *LTBP4* Genotyping

Genomic DNA was extracted from peripheral blood leukocytes using QIAamp DNA Blood Mini Kit (QIAGEN, Germany), following the manufacturer's protocol. SNPs in *SPP1* (rs28357094, rs11730582, and rs17524488) and *LTPB4* (rs2303729, rs1131620, rs1051303, and rs10880) were genotyped by Sequenom MassARRAY iPLEX platform (Sequenom, San Diego, CA, USA), following standard protocols and using primers designed in Sequenom MassARRAY Design 3.1. To enhance differences between alleles, targets were amplified by PCR, reacted with shrimp alkaline phosphatase, and extended with iPLEX primers. After purification on clean resin, products were spotted onto a 384-well SpectroCHIP using MassARRAY RS 1000, analyzed by mass spectrometry on a MassARRAY Analyzer 4 (Sequenom), and called in MassArray Typer 4.0 (Sequenom). Ten percent of samples were duplicated for quality control, and failed reactions were re-genotyped by Sanger sequencing.

### Statistics

Median age at loss of ambulation was estimated and compared using Kaplan-Meier curves and log-rank tests, with ambulant patients censored from analysis. Additionally, Cox proportional hazard regression models were used to assess the contribution of steroid use, *DMD* genotype, and *SPP1*/*LTBP4* SNPs to risk of ambulation loss. For *SPP1*, patients were grouped based on dominant and recessive model. For *LTBP4*, patients were grouped based on recessive model. Data were analyzed in SPSS 22.0 (IBM) and GraphPad Prism 5 (CA, USA), with statistical significance set at *p* < 0.05.

## Results

### Age at Ambulation Loss Stratified by GCs Use and *DMD* Genotype

**Figure 1:**
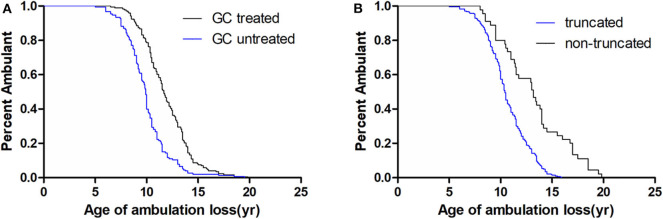
Kaplan–Meier plots of age at loss of ambulation for 326 Duchenne muscular dystrophy patients stratified by **(A)** GCs treatment and **(B)** *DMD* genotype.

Median age at loss of ambulation was 10.50 years for the entire cohort (*n* = 326; Table 1). Strikingly, treatment with GCs significantly delayed ambulation loss to 11.67 years (*n* = 173) from 9.92 years in untreated patients (*n* = 153, *p* < 0.001; Table 1, Figure 1). In addition, patients with non-truncation mutations in *DMD* (*n* = 45, Table S1) lost ambulation 2.75 years later, at median age 13.17 years, than patients with truncation mutations, who lost ambulation at median age 10.42 years (*n* = 281, *p* < 0.001; Table 1, Figure 1).

**Table 1:** Median age at loss of ambulation, stratified by glucocorticoid use and *DMD* genotype.

	***N***	**Median age** **at loss of ambulation, yr**	**KM^†^ Log-Rank *p***
**All patients**	326	10.50	
**Glucocorticoid use**			<0.001^***^
Treated	173	11.67	
Untreated	153	9.92	
***DMD*** **genotype**			<0.001^***^
Truncated mutations	281	10.42	
Non-truncated mutations	45	13.17	

† KM, Kaplan–Meier survival analysis with log-rank comparison.

*** *p < 0.001*.

### *SPP1* and *LTPB4* Genotyping

The minor allele frequency (MAF) for rs28357094, an *SPP1* SNP, was <0.01 (1 in 326), in line with reference Asian populations in the 1000 Genomes project (http://www.1000genomes.org/), and implying that the cohort was homogeneous. In contrast, the MAFs for the *SPP1* SNPs rs17524488 and rs11730582 were 0.40 and 0.37, respectively. All these SNPs were in Hardy Weinberg equilibrium.

The *LTPB4* SNPs rs2303729, rs1131620, rs1051303, and rs10880 are usually co-inherited due to strong linkage disequilibrium across the *LTBP4* locus. Accordingly, the MAFs for these SNPs were 0.44, 0.44, 0.44, and 0.37, respectively, and the frequencies of reconstructed LTBP4 protein haplotypes were 0.56 (VTTT), 0.37 (IAAM), and 0.07 (IAAT). The combined frequency of all other rare haplotypes was <0.01. As with *SPP1, LTBP4* genotypes were consistent with reference Asian populations in the 1000 Genomes project, and were in Hardy Weinberg equilibrium.

### Effect of *SPP1* Polymorphisms

**Figure 2:**
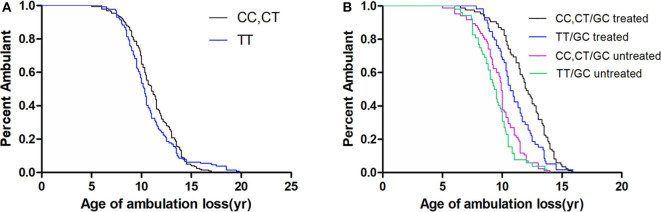
Kaplan–Meier plots of age at loss of ambulation for **(A)** 326 Duchenne muscular dystrophy patients stratified by *SPP1* rs11730582, and for **(B)** patients with truncated *DMD* and stratified by *SPP1* rs11730582 genotype and GCs treatment.

As the MAF of *SPP1* rs28357094 was exceedingly low, we only analyzed the relationship of rs11730582 and rs17524488 to loss of ambulation. As shown in Table 2, In a dominant model, the median age at loss of ambulation was 11.00 years in patients with the CC/CT genotype at rs11730582 (*n* = 197), but 10.33 years in patients with homozygous T alleles (*n* = 129, *p* = 0.272, Figure 2). In a recessive model, ambulation was lost at median age 11.17 years in patients with homozygous C alleles (*n* = 41), and at 10.50 years in patients with the TT/CT genotype (*n* = 285, *p* = 0.769.). In Cox proportional hazard models, the hazard ratio was 0.81 (95% confidence interval 0.64–1.02, *p* = 0.071.) for the CC/CT genotype, 0.42 (95% confidence interval 0.34–0.53, *p* < 0.001) for long-term treatment with GCs, and 0.28 (95% confidence interval 0.19–0.41, *p* < 0.001) for patients with non-truncated *DMD* genotypes, suggesting that the latter two were confounding factors. Among patients treated with GCs and who have truncated *DMD* genotypes, loss of ambulation was delayed by 1.33 years if the rs11730582 genotype was CC/CT (*n* = 85), with median age 12.00 years at loss of ambulation, and hazard ratio 0.63 (95% confidence interval 0.45–0.89, *p* = 0.008). In comparison, median age at loss of ambulation was 10.67 if the rs11730582 genotype was TT (*n* = 59, *p* = 0.006, Figure 2). However, rs11730582 genotypes did not affect loss of ambulation in patients who did not receive GCs, as shown in Table 2.

**Table 2:** Effect of steroid use, *DMD* genotype, and *SPP1* rs11730582 genotype on ambulation loss.

		**Dominant model for C**			**Recessive model for C**		
		**CC/CT**	**TT**		**CC**	**TT/CT**	
All patients	*N*	197	129		41	285	
	Median age at onset, yr	11.00	10.33		11.17	10.50	
	KM ‡Log-Rank *p*			0.272			0.769
	HR ^§^(95% CI^¶^)			0.81 (0.64–1.02)			0.95 (0.69–1.32)
	Cox *p*			0.071			0.774
GCs^†^ treated/truncated *DMD*	*N*	85	59		15	129	
	Median age at onset, yr	12.00	10.67		12.00	11.50	
	KM Log-Rank *p*			0.006^**^			0.771
	HR (95% CI)			0.63 (0.45–0.89)			0.93 (0.54–1.58)
	Cox *p*			0.008^**^			0.776
GCs untreated/truncated *DMD*	*N*	85	52		20	117	
	Median age at onset, yr	9.92	9.33		10.00	9.50	
	KM Log-Rank *p*			0.104			0.148
	HR (95% CI)			0.76 (0.53–1.08)			0.72 (0.44–1.15)
	Cox *p*			0.120			0.169

† GCs, glucocorticoids.

KM, Kaplan–Meier survival analysis with log-rank comparison of median age at loss of ambulation.

§ HR, hazard ratio for SPP1 genotype in a Cox regression model.

¶ CI, confidence interval.

** *p < 0.01*.

On the other hand, the minor GG alleles of *SPP1* rs17524488 did not significantly impact ambulation loss in dominant or recessive models. The hazard ratio was 1.00 (95% confidence interval 0.80–1.26, *p* = 0.984) for the GGGG/GGG genotype, 0.42 (95% confidence interval 0.33–0.53, *p* < 0.001) for patients treated with GCs, and 0.29 (95% confidence interval 0.20–0.42, *p* < 0.001) for patients with non-truncated *DMD* genotypes. In patients with truncated *DMD*, had a GG genotype, and who received GCs therapy, ambulation loss was delayed by 0.50 years, although this effect was not statistically significant. rs17524488 data are listed in Table S2.

### Effect of *LTPB4* Haplotype

**Figure 3:**
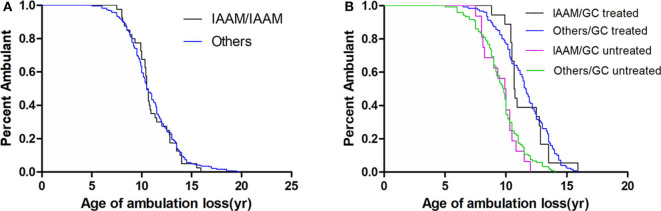
Kaplan–Meier plots of age at loss of ambulation for **(A)** 326 Duchenne muscular dystrophy patients stratified by *LTBP4* haplotype and for **(B)** patients with truncated *DMD* and stratified by *LTBP4* haplotype and GCs treatment.

In a recessive model, the *LTBP4* IAAM haplotype did not significantly affect median age at loss of ambulation (Figure 3). The hazard ratio was 1.06 (95% confidence interval 0.76–1.48, *p* = 0.713) for IAAM/IAAM, 0.42 (95% confidence interval 0.33–0.53, *p* < 0.001) for patients treated with GCs, and 0.29 (95% confidence interval 0.20–0.42, *p* < 0.001) for patients with truncated *DMD*. Statistical differences were not observed in combinations of treatment, *DMD* genotype, and *LTBP4* haplotype (Table S3).

## Discussion

We have now assessed the effects of environmental and genetic factors on the progression of DMD in Chinese patients. Of note, steroids that have anti-fibrosis activity are widely considered to be the most significant environmental factor (33). Indeed, we observed a 1.75-year delay in loss of ambulation in patients treated with GCs, even when administered at doses (~0.5 mg/kg/d) lower than recommended (0.75 mg/kg/d) (34) to minimize side effects.

Different mutations in *DMD* might also contribute to the variability in phenotype severity. For example, Bello et al. (29) and van den Bergen et al. (28) reported that the median age to ambulation loss was significantly different between patients with deletions amenable to exon 44 skipping and patients with other forms of deletions. Such spontaneous skipping restores the *DMD* reading frame, produces some functional dystrophin that may alleviate the disease phenotype and delay ambulation loss by several years, and may occur more frequently in patients with a deletion flanking exon 44, as well as in patients with nonsense mutations, small indel mutations in in-frame exons such as exons 23–42 (27), which encode the functionally dispensable rod domain in dystrophin and are defined by weaker splicing signals. Besides, deletion of exons 3–7 and missense mutations are also associated with milder phenotypes according to previous studies (30–32). As these mutations were too few in our cohort, we combined them into one group of non-truncation mutations as the confounding factor for the further genetic modifiers analysis.

The SNP rs28357094 (-66T/G) in the *SPP1* promoter was previously described as a genetic modifier in DMD (7, 8), the less common G allele was associated with more rapid disease progression, especially in patients treated with GCs (9), implying that this variant may act as a pharmacodynamic biomarker of steroid response, rather than of disease progression itself. However, the frequency of the G allele in rs28357094 was <0.01 in our cohort, in concordance with reference Asian populations from the 1000 Genomes database, for which the minor allele frequency is 0. Hence, meaningful association analysis for this allele was impossible. In light of this, we assessed the effects of the more common *SPP1* promoter SNPs rs11730582 (-443C/T) and rs17524488 (-156G/GG). The minor C allele of rs11730582 was significantly associated with prolonged ambulation in DMD patients treated with GCs in a dominant model. Previous study measured the promoter activity of the−443 C>T polymorphism and found a significantly higher luciferase activity in the pGL3-C construct compared to the pGL3-T construct (35). Moreover,−443 C was also found to be associated with more severe inflammatory disease (12, 36) and rapider tumor progression and metastasis (14, 15, 37), which was not consistent with the manifestation in DMD patients treated with GCs, suggesting that GCs might be a critical factor in the regulation of *SPP1*.

OPN, encoded by *SPP1*, acts as a pro-inflammatory cytokine in muscle, and high OPN expression worsen the phenotype of dystrophin deficiency. Steroids are notable transcriptional regulators of inflammation-related genes including *SPP1*. Multiple potential steroid hormone enhancers were found in the *SPP1* promoter containing vitamin D receptor, glucocorticoid receptor, and estrogen receptor, as well as a putative NF-κB binding site (38). The minor G allele in rs28357094 was reported to drive low basal gene expression, but to elicit a 3-fold increase in expression in response to estrogen, while the T allele elicited abundant baseline expression but was insensitive to estrogen. The insensitivity of the T allele to estrogen (and possibly to NF-κB) is due to the binding of the Sp1 transcription factor near the mediator complex. Given the promoter structure in *SPP1*, GCs may elicit similar effects as estrogen in patients with G alleles. Consequently, increased *SPP1* expression in response to GCs may exacerbate muscle inflammation and produce a worse phenotype (38). Of note, homozygous C alleles in rs11730582 were previously demonstrated to drive significantly more abundant expression of *SPP1* mRNA in melanoma cells compared to either heterozygous CT or homozygous TT genotypes (39). This increased basal expression is due to the binding of the transcription factor c-Myb to−443C but not to−443T alleles. In turn, binding of c-Myb to the minor C allele may prevent a transcriptional response to steroids, as the T allele in rs28357094, a hypothesis that needs to be tested in further studies.

All three *SPP1* promoter SNPs at−66,−156, and−443 have been surveyed simultaneously in several diseases, including ischemic stroke (10), glioma (15), osteoarthritis (12), cervical spondylotic myelopathy (13), and a variety of cancers (11, 14). It had also been proved that the haplotype−443T/-156GG/-66G appear to significantly lower promoter activity in comparison to the other five haplotypes (40). On the other hand, several other surveys of Caucasian patients did not indicate an association between rs28357094 and progression of DMD patients (22, 23), and our own data did not indicate an association between rs17524488 and ambulation loss. Considering the complicated genotype-phenotype correlation between *SPP1* and DMD progression, we speculate that *SPP1* promoter haplotypes, rather than single variants, are more critical drivers of expression and are stronger determinants of interactions between transcription factors and the *SPP1* promoter.

In contrast to several earlier surveys, we did not observe a specific effect of LTBP4 haplotypes on ambulation loss in Chinese patients. Flanigan et al. (22) were the first to report that a homozygous IAAM haplotype delays loss of ambulation in the United Dystrophinopathy Cohort. This result was subsequently confirmed by van den Bergen et al. (23) in patients from five European neuromuscular centers. Similarly, Bello et al. (9) detected a strong association between LTBP4 haplotype and disease progression in a Caucasian cohort, but not in a mixed population. Collectively, these results suggest that the effects of genetic modifiers may vary with ethnic background for differences in allele frequency/haplotype configuration, other environmental factors, standard of care, and phenotype severity. Hence, it is necessary to survey the effects of *LTBP4* haplotypes in other non-Caucasian DMD cohorts.

Limitations in the present study include the low number of patients once stratification is done, and only Chinese patients were enrolled, the associations of *SPP1* rs11730582 in DMD treated with GCs needs to be confirmed in a larger sample size and replicated in other ethnic populations. Secondly, the “non-treatment” group is heterogeneous and includes participants with up to 1 year of GCs treatment. We and other researchers choose the 1-year treatment threshold as the standard of long-term effect of GCs treatment, which is worth further discussing considering GCs has a significant impact on the progress of DMD. Moreover, further studies will be required to address the molecular mechanism of the association of the C allele with less severe phenotype in DMD treated with GCs.

In summary, our data highlight *SPP1* rs11730582 as a genetic modifier of the long-term effect of GCs treatment in Chinese patients with Duchenne muscular dystrophy, but do not indicate a previously reported association between *LTBP4* haplotype and disease progression. Hence, future observational and interventional studies in DMD should adjust for GCs use, *DMD* genotypes, and *SPP1* polymorphisms.

## Data Availability Statement

The full data set has been uploaded to Figshare for permanent storage. doi: 10.6084/m9.figshare.12421064.

## Ethics Statement

The studies involving human participants were reviewed and approved by ICE for Clinical Research and Animal Trial at First Affiliated Hospital of Sun Yat-sen University. Written informed consent to participate in this study was provided by the participants' legal guardian/next of kin.

## Author Contributions

CZ, YZha, and MC designed the study. MC, LW, HZ, YZhu, RH, HL, and JL conducted follow-up. YL and YC analyzed data. All authors were involved in writing and revising the article.

## Conflict of Interest

The authors declare that the research was conducted in the absence of any commercial or financial relationships that could be construed as a potential conflict of interest.
