# Supplementary material for: Genetic Modifiers of Duchenne Muscular Dystrophy in Chinese Patients
Source: Front Neurol. 2020 Jul 29;11:721. doi: 10.3389/fneur.2020.00721 (PMC7403400; doi:10.3389/fneur.2020.00721)
Supplement: Supplementary file 1 [file Table_1.DOC]

Table S1. Distribution of *DMD* genotypes in patients

|  | Number of patients |
| --- | --- |
| **Truncated *DMD* Mutation Type** | 281 |
| out-of-frame deletions | 196 |
| out-of-frame duplication | 24 |
| Nonsense mutations within out-of-frame exons | 44 |
| Splice | 11 |
| Indel mutations out-of-frame exons | 6 |
| **Non-truncated *DMD* Mutation Type** | 45 |
| In-frame deletion/ duplication | 12 |
| Nonsense mutations within in-frame exons | 17 |
| Deletions amenable to skipping exon 44 | 5 |
| Deletion of exons 3–7 | 2 |
| Missense | 6 |
| Indel† mutations within in-frame exons | 3 |

† Indel: insertion or deletion
