# Supplementary material for: Genetic Modifiers of Duchenne Muscular Dystrophy in Chinese Patients
Source: Front Neurol. 2020 Jul 29;11:721. doi: 10.3389/fneur.2020.00721 (PMC7403400; doi:10.3389/fneur.2020.00721)
Supplement: Supplementary file 2 [file Table_2.DOC]

Table S2. Effect of steroid use, *DMD* genotype, and *SPP1* rs17524488 genotype on ambulation loss

|  | | **Dominant model for GG** | | | **Recessive model for GG** | | |
| --- | --- | --- | --- | --- | --- | --- | --- |
|  |  | **GGGG/GGG** | **GG** |  | **GGGG** | **GGG/GG** |  |
| **All Patients** | **n** | **211** | **115** |  | **52** | **274** |  |
|  | **Median Age**  **at LoA, yr** | **10.50** | **10.67** |  | **10.50** | **10.50** |  |
|  | **KM**‡ **Log-Rank** *p* |  |  | **0.983** |  |  | **0.749** |
|  | **HR**§**(95% CI**¶**),**  **Cox p** |  |  | **1.00(0.80-1.26),**  **0.984** |  |  | **0.95(0.71-1.29),**  **0.755** |
| **GCs**† **Treated/**  **Truncated *DMD*** | **n** | **94** | **50** |  | **28** | **116** |  |
|  | **Median Age**  **at LoA, yr** | **11.42** | **11.92** |  | **11.58** | **11.50** |  |
|  | **KM Log-Rank** *p* |  |  | **0.478** |  |  | **0.846** |
|  | **HR (95% CI),**  **Cox p** |  |  | **1.13(0.80-1.59), 0.490** |  |  | **1.04(0.69-1.58), 0.850** |
| **GCs Untreated/**  **Truncated *DMD*** | **n** | **86** | **51** |  | **19** | **118** |  |
|  | **Median Age**  **at LoA, yr** | **9.50** | **10.00** |  | **9.50** | **9.83** |  |
|  | **KM Log-Rank** *p* |  |  | **0.173** |  |  | **0.457** |
|  | **HR(95% CI),**  **Cox p** |  |  | **1.26(0.89-1.79), 0.192** |  |  | **1.20(0.73-1.95), 0.474** |

† GCs, glucocorticoids;

‡ KM, Kaplan–Meier survival analysis with log-rank comparison of median age at loss of ambulation;

§ HR, hazard ratio for *SPP1* genotype in a Cox regression model;

¶ CI, confidence interval.
