# Supplementary material for: Genetic Modifiers of Duchenne Muscular Dystrophy in Chinese Patients
Source: Front Neurol. 2020 Jul 29;11:721. doi: 10.3389/fneur.2020.00721 (PMC7403400; doi:10.3389/fneur.2020.00721)
Supplement: Supplementary file 3 [file Table_3.DOC]

Table S3. Effect of steroid use, *DMD* genotype, and *LTBP4* haplotype genotype on ambulation loss

|  | | **Recessive model for IAAM** | | |
| --- | --- | --- | --- | --- |
|  |  | **IAAM/IAAM** | **Others** |  |
| **All Patients** | **n** | **40** | **286** |  |
|  | **Median Age**  **at LoA, yr** | **10.50** | **10.50** |  |
|  | **KM**‡ **Log-Rank** *p* |  |  | **0.706** |
|  | **HR**§**(95% CI**¶**),**  **Cox p** |  |  | **1.06(0.76-1.48),**  **0.713** |
| **GCs**† **Treated/**  **Truncated *DMD*** | **n** | **18** | **126** |  |
|  | **Median Age**  **at LoA, yr** | **10.67** | **11.58** |  |
|  | **KM Log-Rank** *p* |  |  | **0.960** |
|  | **HR(95% CI),**  **Cox p** |  |  | **1.01(0.61-1.69), 0.961** |
| **GCs Untreated/**  **Truncated *DMD*** | **n** | **16** | **121** |  |
|  | **Median Age**  **at LoA, yr** | **9.92** | **9.83** |  |
|  | **KM Log-Rank** *p* |  |  | **0.676** |
|  | **HR(95% CI),**  **Cox p** |  |  | **1.11(0.66-1.88), 0.687** |

† GCs, glucocorticoids;

‡ KM, Kaplan–Meier survival analysis with log-rank comparison of median age at loss of ambulation;

§ HR, hazard ratio for *LTBP4* genotype in a Cox regression model;

¶ CI, confidence interval.
